# Investigating the cause of cardiovascular dysfunction in chronic kidney disease: capillary rarefaction and inflammation may contribute to detrimental cardiovascular outcomes

**DOI:** 10.1007/s00395-024-01086-6

**Published:** 2024-10-30

**Authors:** Siavash Beikoghli Kalkhoran, Maryna Basalay, Zhenhe He, Pelin Golforoush, Tayeba Roper, Ben Caplin, Alan D. Salama, Sean M. Davidson, Derek M. Yellon

**Affiliations:** 1 The Hatter Cardiovascular Institute, University College London & UCL Hospital, 67 Chenies Mews, London, WC1E 6HX UK; 2 Centre for Kidney and Bladder Health, Royal Free Hospital, University College London, London, England, UK

**Keywords:** Chronic kidney disease, Heart, Ischaemia, Reperfusion injury, Inflammation

## Abstract

Myocardial ischemia–reperfusion (IR) injury is a major cause of morbidity and mortality in patients with chronic kidney disease (CKD). The most frequently used and representative experimental model is the rat dietary adenine-induced CKD, which leads to CKD-associated CVD. However, the continued intake of adenine is a potential confounding factor. This study investigated cardiovascular dysfunction following brief adenine exposure, CKD development and return to a normal diet. Male Wistar rats received a 0.3% adenine diet for 10 weeks and normal chow for an additional 8 weeks. Kidney function was assessed by urinalysis and histology. Heart function was assessed by echocardiography. Sensitivity to myocardial IR injury was assessed using the isolated perfused rat heart (Langendorff) model. The inflammation profile of rats with CKD was assessed via cytokine ELISA, tissue histology and RNA sequencing. Induction of CKD was confirmed by a significant increase in plasma creatinine and albuminuria. Histology revealed extensive glomerular and tubular damage. Diastolic dysfunction, measured by the reduction of the E/A ratio, was apparent in rats with CKD even following a normal diet. Hearts from rats with CKD had significantly larger infarcts after IR injury. The CKD rats also had statistically higher levels of markers of inflammation including myeloperoxidase, KIM-1 and interleukin-33. RNA sequencing revealed several changes including an increase in inflammatory signaling pathways. In addition, we noted that CKD induced significant cardiac capillary rarefaction. We have established a modified model of adenine-induced CKD, which leads to cardiovascular dysfunction in the absence of adenine. Our observations of capillary rarefaction and inflammation suggest that these may contribute to detrimental cardiovascular outcomes.

**Supplementary Information:**

The online version contains supplementary material available at 10.1007/s00395-024-01086-6.

## Introduction

The prevalence of chronic kidney disease (CKD) is estimated at 9–13% in developed countries and is increasing [78]. A wealth of epidemiological evidence suggests that both dialysis and non-dialysis-dependent CKD represent independent risk factors for cardiovascular death [10]. Indeed, half of the deaths in advanced CKD patients are of cardiovascular causes, with myocardial infarction (MI) being the most frequent event [59]. Conversely, more than 30% of patients who experience myocardial infarction do so on a background of pre-existing CKD, and CKD is a strong independent predictor of worse cardiovascular and renal outcomes and mortality [20, 83].

Numerous animal models have been developed to study CKD-associated cardiovascular diseases (CVD) [49, 67]. However, these models often have limitations and may not fully recapitulate the pathologies seen in human patients. For instance, surgical models of CKD involve the removal of a substantial portion of the kidneys, which differs from human CKD where the kidney damage may have an inflammatory component and develops at a slower pace [32, 45]. Importantly, unlike human patients, animal models of CKD do not often lead to CVD [13, 34, 85]. The adenine-induced model is most similar to human CKD. In this model, accumulation of dietary adenine in renal tubules mediates slow but progressive inflammatory CKD, leading ultimately to cardiac complications [4, 15, 89]. The first model that was developed of adenine-induced CKD in rats used a high adenine (0.75%) diet, which caused significant tubular damage and accumulation of crystals in the renal tubules. The rats also exhibited a substantial reduction in body weight and a rapid decline in renal function after only 6 days [90]. Diwan et al. reported similar findings, showing that a 0.75% adenine diet caused acute kidney toxicity as evidenced by early glomeruli sclerosis and tubular atrophy. Furthermore, the rats had to be euthanized at 6 weeks due to drastic weight loss. These aspects made the model deviate from the slow onset of CKD as seen in human patients [15]. To slow disease progression and prevent rapid weight loss, lower doses consisting of 0.25% or 0.3% dietary adenine have been used, which avoid the rapid induction of CKD to facilitate renal and cardiac pathologies [15, 87]. The renal pathologies of the lowered doses of adenine include disrupted creatinine clearance, proteinuria, glomerulopathy, tubulopathy, and systemic inflammation [15, 87]. The lower dose of adenine also induces cardiovascular pathologies including raised systolic blood pressure (BP), diastolic dysfunction, maladaptive vascular function and systemic inflammation [15, 40, 87], which closely mimic the pathologies seen in human patients [11, 64]. Another potential limitation of most published models of adenine-induced CKD is that adenine is administered throughout the entire study protocol [4, 15]. Considering that adenine can cause continuous kidney injury [42], oxidative stress [38], and metabolic disorders [7, 8], we thought it relevant to follow the adenine diet with a “washout” period to ensure that the outcomes observed are due to the underlying CKD and not to adenine-induced acute toxicity.

In this study, we adapted an established model of adenine-induced CKD [15, 87]. We included an extended treatment with a diet containing 0.3% adenine for 10 weeks, followed by a washout period of 8 weeks to remove the potential confounding impact of continued adenine presence. We aimed to assess the development of both cardiac and kidney dysfunction and identify potential mechanisms that connect CKD to cardiac dysfunction and sensitivity to ischemia and reperfusion (IR) injury. To address these aims, we assessed systemic inflammation and changes in capillary density, both of which have been suggested to contribute to the pathogenesis of cardiac and renal dysfunction in adenine-induced CKD [9, 71]. We also conducted RNA sequencing of the heart to better understand the key genes and signaling pathways that contribute to the development of cardiac pathologies in our model of adenine-induced CKD.

Our results demonstrate that compromised kidney function persists despite the change of adenine diet and ultimately leads to systemic inflammation, cardiac dysfunction, capillary rarefaction and increased sensitivity to IR injury.

## Methods

### Animals

All animal experiments were conducted in accordance with the United Kingdom Animals (Scientific Procedures) Act 1986 Amendment Regulations 2012. The procedures performed in this study were part of the project license PPL 70/8556, approved by the Animal Welfare and Ethical Review Board of UCL and the Home Office.

### Experimental groups and procedures

**Fig. 1:**
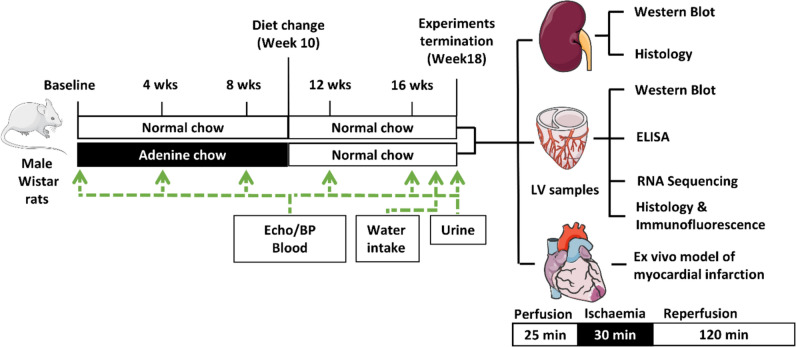
Experimental details and timeline

As shown in Fig. 1, 8- to 10-week-old male Wistar rats were randomly divided into two groups receiving either normal chow or 0.3% adenine-enriched chow (referred to as adenine group in this article; SSNIFF S9460-E012) for 10 weeks. All groups then received normal chow for a further 8 weeks. Rats’ weight and food consumption were measured weekly and water consumption was measured at the final week prior to the termination of the experiments. Plasma and urinary creatinine were assessed by the biochemistry department of the Royal Free Hospital using the RX Daytona + clinical chemistry analyzer. Urinary albumin was quantified via a rat albumin ELISA (Cambridge bioscience) at the end of the experiments. Tissues were harvested and analyzed as described below.

Timecourse of the experiment indicating the period of adenine-enriched diet administration, sampling frequency, and the downstream assays. LV: left ventricle, Echo: echocardiography, BP: blood pressure.

An isolated, perfused ex vivo rat heart (Langendorff) model was used to determine the effects of adenine-induced CKD on myocardial infarct size following IR. Following the establishment of deep anesthesia by inhaled isoflurane (4%), the hearts were excised, the aorta cannulated, and mounted on a purpose-built Langendorff system. Hearts were then retrogradely perfused with Krebs–Henseleit buffer [37] for 25 min to establish a stable state before being subjected to 30 min of global ischemia and 120 min of reperfusion. Three hearts were omitted from the analysis based on our previously established exclusion criteria [3]. Of these, one heart was excluded from each of the control and adenine groups due to ventricular fibrillation for more than 3 min. One heart in the adenine group was also excluded due to an initial flow rate of less than 10 ml/min. The hearts were then frozen, sliced, and incubated in 1% triphenyltetrazolium chloride (TTC) stain at 37 °C for 20 min. Slices were fixed in formalin overnight and imaged the next day. Infarct size was analyzed using the Image J software. Infarct size was adjusted to the weight of each slice and was expressed as a percentage of the area at risk of infarction (IS/AAR%). The entire area of each slice representing both ventricles of the myocardium was selected as AAR for the purpose of analyzing the infarct size.

### Blood glucose

Blood glucose was tested monthly via venous sampling from the rat tail vein using the Accu-Chek Mobile blood glucose meter (Roche).

### Blood pressure

Heart rate, systolic blood pressure (SBP), and diastolic blood pressure (DBP) were measured using the tail-cuff non-Invasive blood pressure system for rodents (Panlab Harvard apparatus) in anesthetized animals. 4% Isoflurane delivered with oxygen (1 L/min) was used for the induction of anesthesia, followed by the reduction of Isoflurane concentration to 2% to maintain anesthesia.

### Echocardiography

Cardiac ultrasound was performed in rats using a GE Vivid-i portable ultrasound machine (GE Medical Systems Ultrasound Israel Ltd.). All measurements were performed under anesthesia as described above. Imaging was conducted by initially locating the heart using long-axis B mode so that both the cardiac apex and the aortic valve were visualized. Then, the following measurements were obtained (in mm) in diastole and systole using M mode at the level of papillary muscles: interventricular septum, LV internal diameter in diastole and systole, and LV posterior wall. We then visualized the short axis of the LV at the same level by turning the probe perpendicular to its initial position, and the same measurements were obtained again. Using these measurements, LV fractional shortening and the ejection fraction were automatically calculated. In addition, in B mode, the cine loops of the long axis of the LV were recorded. In these loops, the diameter and the length of LV in systole and diastole were measured (in mm). From these measurements, end-diastolic volume and end-systolic volume were calculated manually. To evaluate the diastolic function of the LV, the mitral flow was first located from the long axis view, using color Doppler mode. The probe was placed at the maximal visual mitral flow velocity, and pulse wave Doppler was used to measure E and A peaks.

### Histology and immunofluorescence

To evaluate the adenine-associated cardiac and renal damage, kidneys and LV transverse slices were fixed in 4% paraformaldehyde overnight and were subsequently placed in 70% ethanol. 10 µm sections were stained with Haematoxylin and Eosin (H and E) and Masson’s trichome staining, to assess inflammation and fibrosis respectively. Picrosirius red staining was also performed to further assess collagen content and fibrosis in the heart. Picrosirius red staining was conducted according to the manufacturer’s instructions (Abcam-AB245887). Vascular calcification was assessed by Von Kossa staining [5]. Renal sections were stained with Periodic Acid Schiff (PAS). The glomerular damage score was determined by randomly choosing 25 glomeruli at × 40 magnification and assessing the presence or absence of damage. Tubular dilatation was determined by estimating the percentage of tubular dilation in each high-power field and assigning a score using the following categories: 0 (0%), 1(1–10%), 2 (25–50%), 3 (50–75%) and 4 (> 75%).

Immunofluorescent staining was used to quantify the extent of white blood cell infiltration in the hearts. Slides with paraffin-embedded heart tissues were initially dewaxed and then rehydrated in Histo-clear solution (2 × 5 min), 100% ethanol (2 × 10 min), 80% ethanol (10 min), and dH_2_O (5 min). The slides were then boiled in sodium citrate solution containing tri-sodium citrate (11.4 µM) and tween-20 (0.05%) at pH 6.0. The slides were then cooled before being washed three times with PBS-tween (PBS-T; 0.1%). Heart sections were then blocked in 10% goat serum for 30 min and were incubated with rabbit anti-CD45 (Proteintech-20103-1-AP; 1:200) overnight at 4 °C. Sections were then washed twice with PBS-T (0.1%) and incubated with Alexa fluor 546-conjugated secondary antibody (Thermo-A-11035; 1:400). Sections were then washed in PBS-T (0.1%) (3 × 4 min) and imaged using the 543 nm HeNe laser line of a Leica SP3 confocal microscope. Four representative images were captured from each slide. The number of CD45-positive cells, localized in the extracellular lumen, was counted in a blinded fashion for each image.

To visualize capillary density in the hearts, slides were initially dewaxed in Histo-clear solution (2 × 5 min). The slides were then blocked with 5% bovine serum albumin in Hanks buffer containing NaCl (140 mM), KCl (5 mM), CaCl_2_ (1 mM), MgSO_4_ (0.4 mM), MgCl_2_ (0.5 mM), Na_2_HPO_4_ (0.3 mM), KH_2_PO_4_ (0.4 mM), D-Glucose (6 mM), and NaHCO_3_ (4 mM) for 1 h. Sections were then stained with wheat germ agglutinin (WGA) (Thermo W11261, 5 µg/ml) and Hoechst (Invitrogen H3570; 1:2000) in the blocking buffer for 30 and 5 min, respectively. To compare the capillary density between the two treatment groups, 5 fields of view were captured. In this study, the number of capillaries was counted in a blinded fashion. However, semi-automated tools have also been developed to assess capillary rarefaction in the heart [16].

### ELISA

To assess the state of circulating inflammatory markers in the adenine group vs. control, we quantified the levels of 13 different rat cytokines (Biolegend 741395) at weeks 11 and 18. We also assessed the levels of myeloperoxidase (MPO; Hoelzel AE33085RA), Kidney injury molecule-1 (KIM-1) (Ray Biotech ELR-TIM1-1), and NT-pro brain natriuretic peptide (NT-proBNP; antibodies A74911) and indoxyl sulfate (AMS BIO; AMS.E02I0039) at week 18. These studies were conducted by ELISA and according to the manufacturer’s instructions.

### Western blot

The protein levels of NLRP3, ANGPT1, GSDMD, and citrate synthase were assessed via Western blot analyses of kidney and LV tissues using the following antibodies NLRP3 (Abcam 270449; 1:1000), GSDMD (Cell signaling 397545; 1:1000), ANGPT1 (Proteintech 27093-1-AP;1:1000), and citrate synthase (Proteintech 16131-1-AP;1:1000). Samples were run on a 10% NuPAGE gel (Invitrogen NP0301BOX) at 90 v for 30 min and 120 v for 2 h. Protein transfer was then conducted using Immobilon^®^-FL polyvinylidene difluoride membrane (Merck) at 120v for 1 h and 20 min. Membranes were then blocked in 5% milk/PBS-T for 1 h at room temperature. The membranes were then incubated with primary antibodies overnight. They were then washed 3 × 5 min in PBS/T and further incubated with secondary antibodies (anti-rabbit 800 CW (1:15,000) or anti-mouse 680LT (1:20,000), Li-Cor) for 1 h at room temperature in dark. Membranes were then washed again for 3 × 5 min in PBS/T and imaged using the Odyssey^®^ Infrared Imaging System. Protein level was analyzed by densitometry using Image Studio™ version 5.0.

### RNA sequencing

Total mRNA was extracted from 30 mg LV pieces using the RNA-easy mini kit (Qiagen 74104). The RNA content and quality were assessed using Thermo-Nanodrop Lite. The KAPA Stranded mRNA hyperprep was conducted as described previously [6] and samples were sequenced using a NextSeq 2000 using a 56 bp paired-end run in collaboration with UCL genomics. Run data were converted to fastq files using Illumina's bcl2fastq Conversion Software. Normalization, modeling, and differential gene expression analysis were conducted using IDEP2 online software (http://bioinformatics.sdstate.edu/idep96/) [23]. We also conducted enrichment analysis using the “Hallmark” gene sets as described previously [48]. Normalized read counts were used to compare gene expression between treatment groups.

### Statistics

Data are expressed as mean ± standard deviation of the mean (SD). Two-way ANOVA with Šidák post-test was used to assess the effect of adenine-induced CKD at different time points. The outputs for individual tests are given in Supplementary Table 1. Student’s *t* test or Man–Whitney *U* test was used to assess differences between two groups when data were normally distributed or non-parametric, respectively. *P* value ≤ 0.05 was considered significant. Data was analyzed using GraphPad Prism version 10.

## Results

### General characteristics of rats receiving adenine-enriched diet

**Fig. 2:**
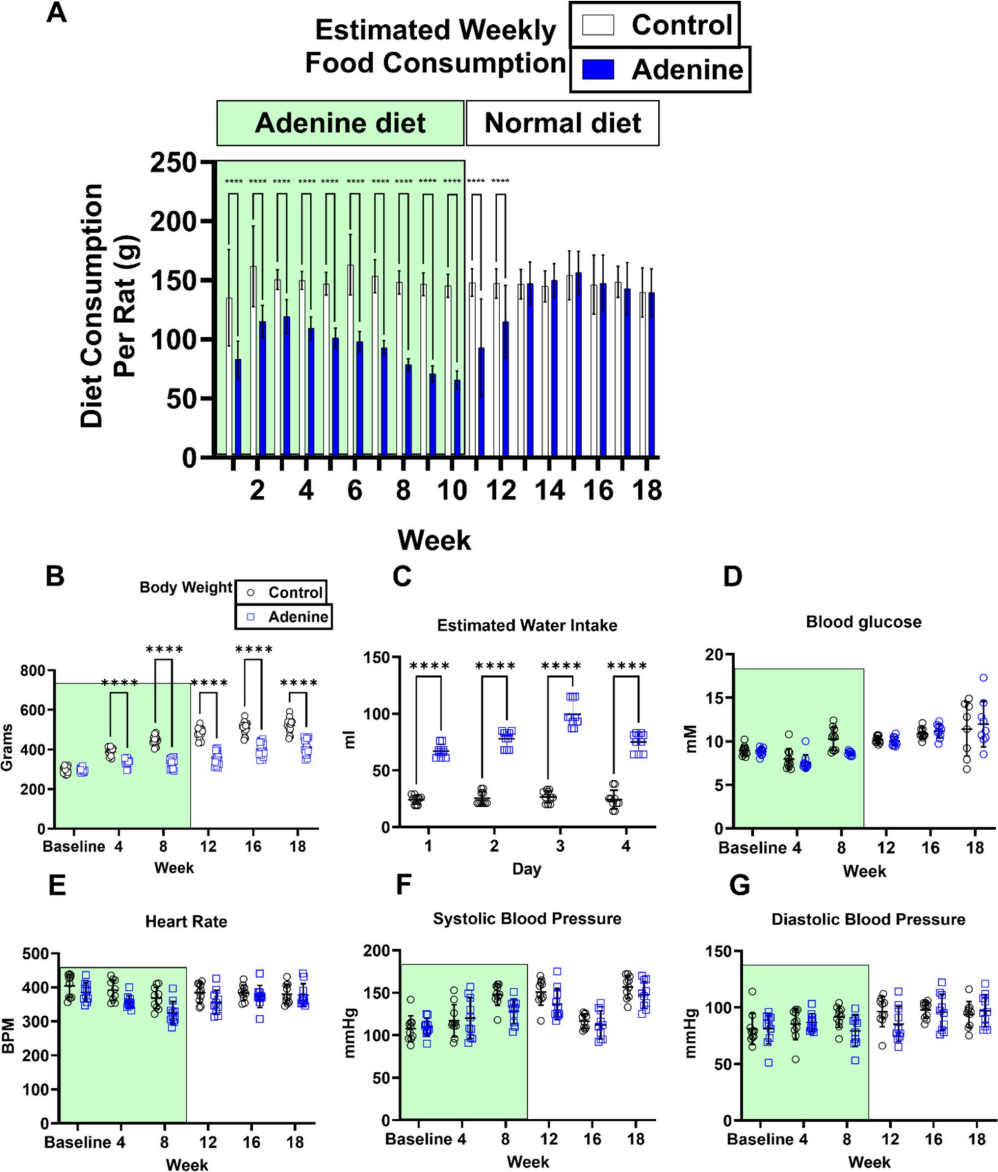
Physical and biochemical characteristics of rats receiving adenine or normal chow. Panel **A** shows the food intake of rats on normal chow (Control) and adenine diet (Adenine) (*n* = 16 per group, ****P < 0.0001 vs. Control). Panel **B** shows body weights throughout the study (*n* = 16 per group, ****P < 0.0001 vs. Control). Panel **C** shows water intake during week 17 (*n* = 10, ****P < 0.0001 vs. Control). Panels **D**–**G** depict the comparison of blood glucose (D; *n* = 10, *P < 0.05 vs. Control), heart rate (E; *n* = 10, **P < 0.01 vs. Control), SBP and DBP (F, G, respectively; *n* = 10, *P < 0.05 vs. Control). All analyses were conducted using two-way ANOVA with Šidák post-test. (Data are expressed as mean ± SD)

Wistar rats were fed either normal chow for 18 weeks (“Control”) or adenine-enriched diet for 10 weeks followed by a further 8 weeks on normal chow (“Adenine”) (Fig. 2A). The physical and biochemical characteristics of both groups of rats are given in Fig. 2 and Table 1. The rats in the adenine group initially ate less than those in the control group (Fig. 2A), but this gradually normalized over 3 weeks following the return to normal chow at week 10. Rats from the adenine group also had a significantly lower body weight in comparison to the control throughout the study (Fig. 2B). Toward the end of the experiment, rats on the adenine diet drank significantly more water in comparison to the rats in the control group (Fig. 2C). Despite these changes, there were no consistent alterations in blood glucose, heart rate, SBP or DBP in either group throughout the study (Fig. 2D–G).

**Table 1:** Physical parameters of different organs in the control versus the adenine group

	Treatment groups mean ± SD (*N* number)		*P* value
	Control	Adenine	
Heart weight	1.15 ± 0.1 (*n* = 10)	1.27 ± 0.17 (*n* = 9)	0.09
Tibia length	5.67 ± 0.21 (*n* = 9)	5.60 ± 0.16 (*n* = 10)	0.44
Heart/Tibia ratio	0.21 ± 0.02 (*n* = 9)	0.23 ± 0.03 (*n* = 9)	0.08
Right kidney weight	1.21 ± 0.13 (*n* = 10)	1.24 ± 0.27 (*n* = 10)	0.80
Left kidney weight	1.20 ± 0.15 (*n* = 10)	1.45 ± 0.25*(*n* = 10)	0.01
Average kidney weight to tibia	0.43 ± 0.04 (*n* = 9)	0.48 ± 0.08 (*n* = 10)	0.12
Lung weight	1.66 ± 0.1 (*n* = 6)	1.76 ± 0.17 (*n* = 6)	0.28

* indicates where there was a significant difference

No significant differences were observed between the two groups in terms of heart and lung weight (Table 1). The total kidney weight was also similar between the two groups. Overall, apart from the body weight, no substantial differences in physical and biochemical parameters were observed between different treatment groups.

### Renal dysfunction persists following the change of the adenine diet

**Fig. 3:**
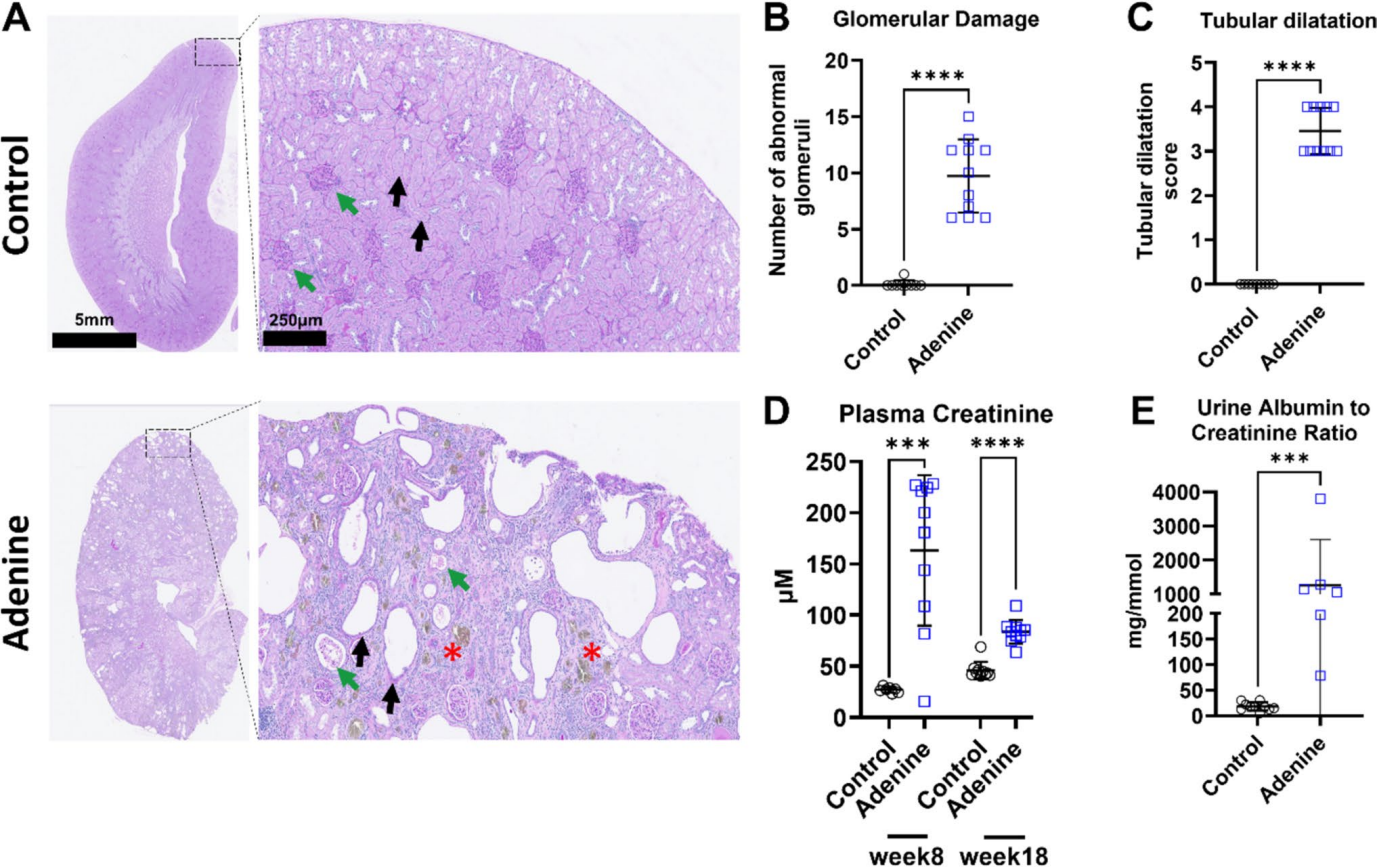
Structural and physiological changes of kidneys associated with dietary adenine intake. In panel **A** the H&E staining of kidneys from rats fed with 0.3% adenine diet indicates glomerular damage, shown by the green arrows and quantified in (**B**)**,** and tubular dilatation, shown by black arrows and quantified in (**C**)**,** (*n* = 9 for control and *n* = 11 for adenine, ****P < 0.0001, Student's *t* test). Sites of crystal deposition are marked with red asterisks. Panel **D** shows the increase in plasma creatinine at week 8 (*n* = 7 for control vs. *n* = 10 for adenine, ***P < 0.001, Student's *t* test) and week 10 *n* = 10, ****P < 0.0001, Student's *t* test), whereas **E** depicts the increase in urinary albumin-to-creatinine ratio in rats from the adenine group (*n* = 10 for control vs. *n* = 6 for adenine, ***P < 0.001, Student's *t* test). (Data are expressed as mean ± SD)

Histological examination of the kidneys from rats on the adenine diet revealed overt signs of chronic glomerular and tubulointerstitial damage as well as crystalline tubular deposits throughout the kidneys in comparison to the rats receiving normal chow. There was a higher percentage of abnormal glomeruli (9.7 ± 3.2 vs. 0.1 ± 0.3% in the adenine vs. control group, *P* < 0.0001, Fig. 3A, B) and tubular damage with a higher tubular dilatation score (3.5 ± 0.5 vs. 0.0 in the adenine vs. control group, *P* < 0.0001, Fig. 3A, C). Rats from the adenine group exhibited pronounced CKD as evidenced by a significant increase in their serum creatinine compared to the control group prior to the wash-out period at week 8 (163.3 ± 73.6 vs. 27.1 ± 2.71 µM in the adenine vs. control group, respectively, *P* < 0.0002, Fig. 3D) and following wash-out period at week 18 (83.8 ± 11.6 vs. 46.2 ± 8.3 µM in the adenine vs. control group, respectively, *P* < 0.0001, Fig. 3D). Rats from the adenine group also had a significant increase in their urinary albumin-to-creatinine ratio (1261.0 ± 1343.0 vs. 18.4 ± 7.9 mg/mmol in the adenine vs. control group, respectively, *P* = 0.0002, Fig. 3E) at week 18. Overall, these data show that kidney dysfunction still persists in rats 8 weeks after the removal of dietary adenine but exhibit lower signs of kidney injury.

### Rats from the adenine group develop cardiac dysfunction

**Fig. 4:**
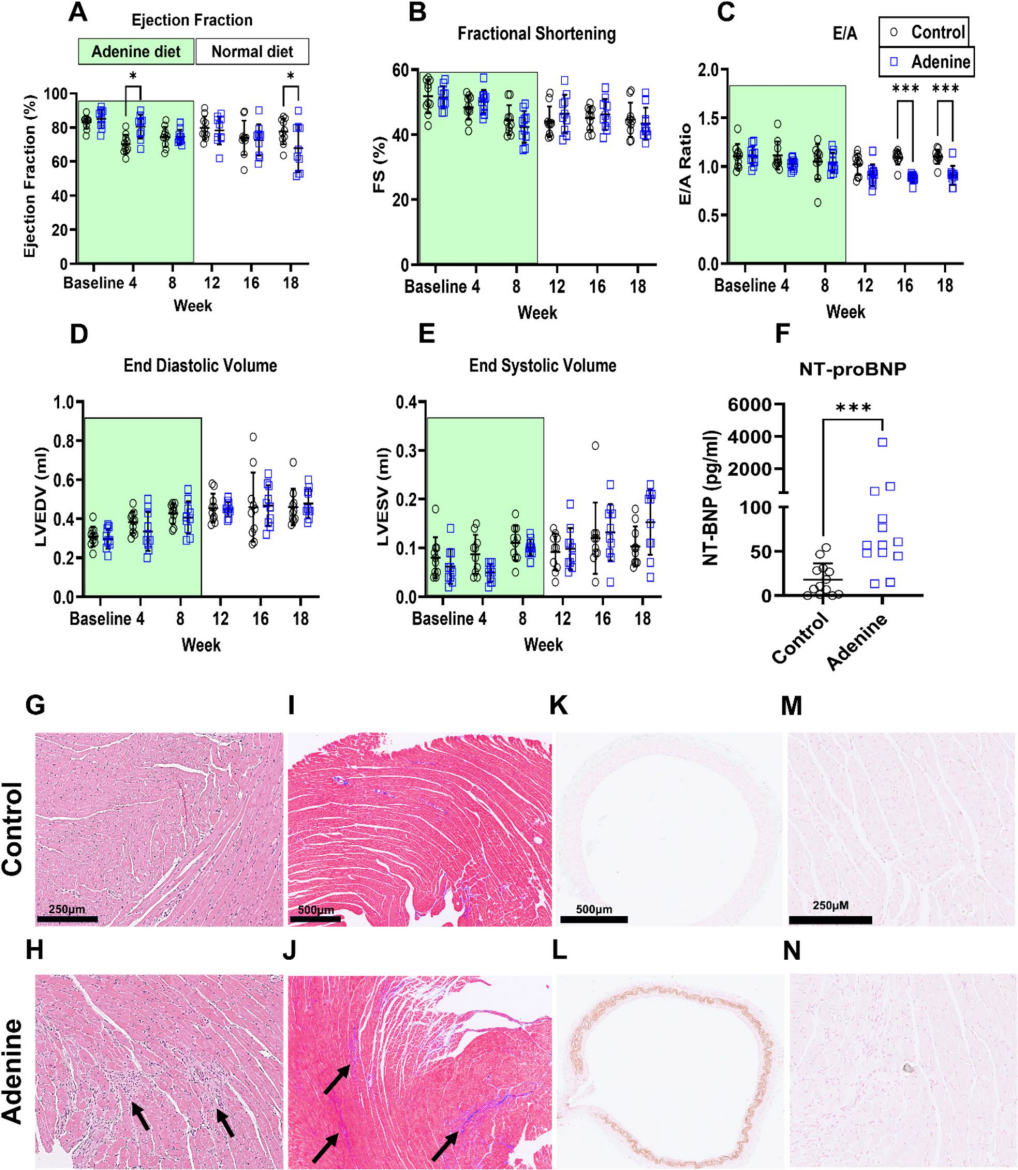
Changes in cardiac function and anatomy. Panels **A** and **B** show the changes in ejection fraction and fractional shortening respectively, (*n* = 10, *P < 0.05, two-way ANOVA with Šidák post-test). The change in the E/A ratio is depicted in **C** whereas the changes in end-diastolic volume and end-systolic volume are shown in (**D**) and (**E**), respectively (*n* = 10, ***P < 0.001, two-way ANOVA with Šidák post-test). The level of NT-proBNP in the plasma is shown in (**F**) (*n* = 13, ***P < 0.001, Mann–Whitney *U* test). Panels **G** and **H** depict the H and E staining of the LV. Black arrows indicate sites of inflammation where there are accumulations of polymorphonuclear cells. **I** and **J** show Masson’s trichome staining of the LV. Areas with shaded purple color represent fibrotic regions and are marked with black arrows. Panels **K** and **L** show von Kossa staining of the calcified rat aorta, whereas **M** and **N** show the same staining of the calcified capillaries in the hearts of the adenine group. (Data are expressed as mean ± SD)

Adenine consumption in rats has been shown to cause cardiac dysfunction [15]. To address the effects of adenine-induced CKD on the heart, we assessed cardiac function by echocardiography. We observed a significant decrease in ejection fraction at 18 weeks in the adenine versus control groups (67.9 ± 13.8 vs. 77.6 ± 7.4%, in the adenine vs. control group, respectively, *P* < 0.03, Fig. 4A). There were also signs of diastolic dysfunction in the adenine group as evident by the decrease in E/A ratio at 16 (0.9 ± 0 vs. 1.1 ± 0.1 in the adenine vs. control group, *P* < 0.0001, Fig. 4C) and 18 weeks (0.9 ± 0.1 vs. 1.1 ± 0.1 in the adenine vs. control group, *P* < 0.0006, Fig. 4C). No differences were present in fractional shortening (Fig. 4B), end-diastolic volume (Fig. 4D) or end-systolic volume (Fig. 4E) between the two groups. Plasma NT-proBNP was significantly elevated in rats with CKD (433.8 ± 999.2 pg/ml vs. 17.9 ± 18.2 in the adenine vs. control group, *P* < 0.0002, Fig. 4F). There was also evidence of local LV inflammation as shown by the accumulations of polymorphonuclear cells in the LV tissue of rats from the adenine group (Fig. 4G, H). In addition, both Masson’s trichrome and Picrosirius red staining confirmed that fibrosis is increased in the hearts of these rats (Fig. 4I, J and Supplementary Fig. 1). We also observed vascular calcification in the aorta (Fig. 4K, L) and small capillaries in the LV tissue (Fig. 4M, N) in rats from the adenine group. These results demonstrate that the cardiac function deteriorated due to kidney dysfunction thus indicating the presence of pathological kidney–heart cross-talk in the absence of dietary adenine [63].

### Systemic and local inflammation in rats fed with an adenine diet

**Fig. 5:**
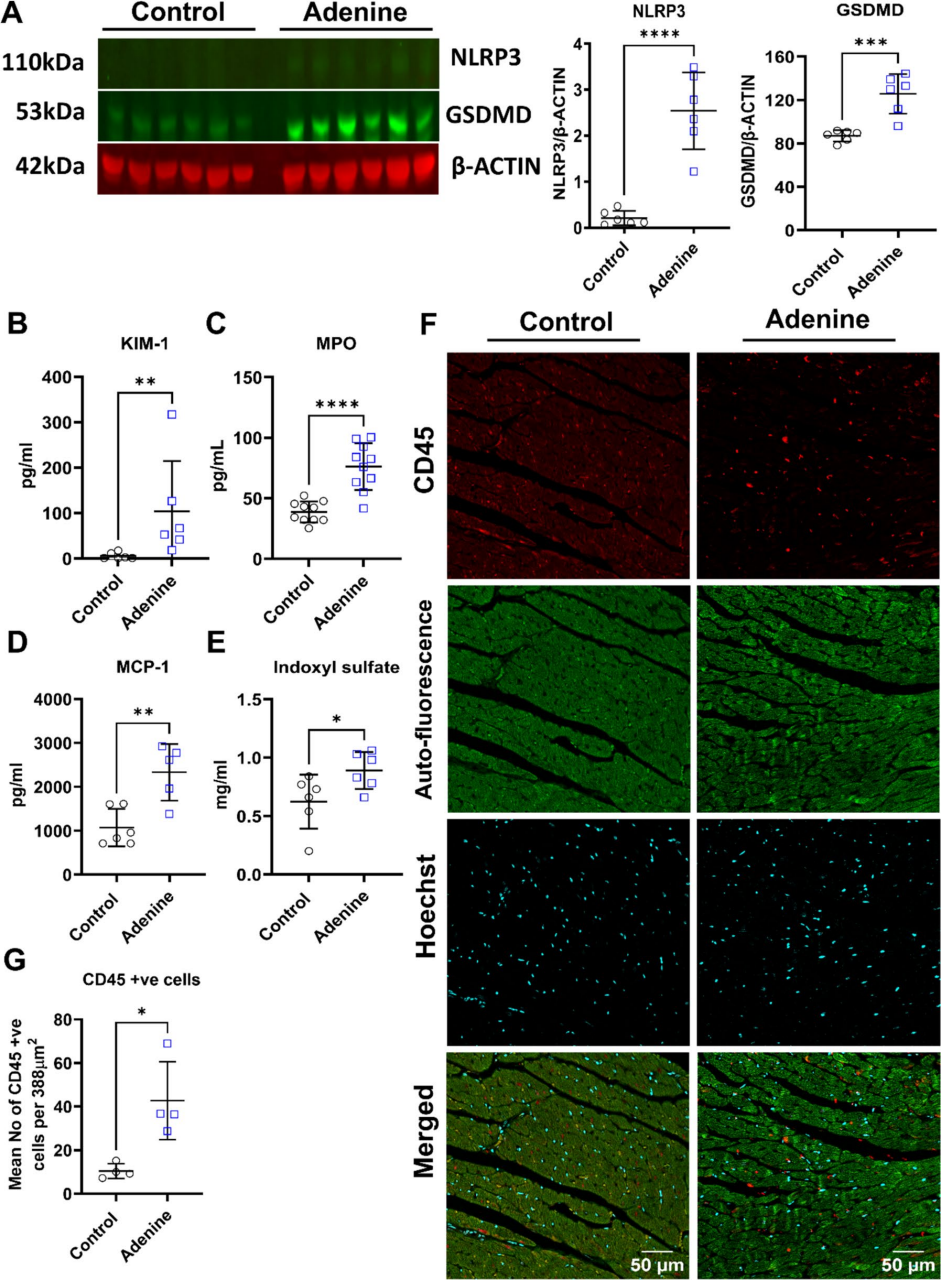
Inflammation in the heart, kidney and circulation of the adenine group. Panel **A** shows the level of markers of pyroptosis including NLRP3 and GSDMD (*n* = 6, ****P < 0.0001 and ***P < 0.001, Student’s *t* test). The plasma level of KIM-1 (*n* = 6, **P < 0.01, Mann–Whitney *U* test.), MPO (*n* = 10 for each group, ****P < 0.0001, Student’s *t* test), and MCP-1 (*n* = 6 for control vs. *n* = 5 for adenine, Student's *t* test) are depicted in panels (**B**–**D**)**.** The level of indoxyl sulfate is shown in (**E**) (*n* = 6, *P* < 0.05, Student’s *t* test). Infiltration of CD45-positive immune cells in the extracellular regions of LV of adenine-fed rats is demonstrated in (**F**) and quantified in (**G**) (*n* = 4, *P < 0.05, Student’s *t* test). (Data are expressed as mean ± SD)

The assessment of kidneys in rats receiving adenine indicated an increase in the protein levels of NLRP3 (2.5 ± 0.8 vs. 0.2 ± 0.1 arbitrary units in the adenine vs. control group, *P* < 0.0001, Fig. 5A) and full-length GSDMD (125.7 ± 18.2 vs. 87.0 ± 5.2 arbitrary units in the adenine vs. control group, *P* = 0.0005, Fig. 5A). However, these proteins were not detectable in the hearts of rats from either group (data not shown). Assessment of circulating inflammatory markers showed that the level of KIM-1 (103.9 ± 45.1 vs. 5.8 ± 7.0 pg/ml in the adenine vs. control group, *P* < 0.002, Fig. 5B) and myeloperoxidase (MPO) (76.2 ± 19.4 vs 38.7 ± 8.6 pg/ml in the adenine vs. control group, *P* < 0.0001, Fig. 5C) was significantly higher in the adenine group. The multiplex assay showed that the levels of two circulating inflammatory cytokines (See Supplementary Figs. 2A–M and 3A–L) were significantly altered at week 11 and/or at the end of the experiment. These were IL-33 (507.5 ± 395.5 vs. 161.8 ± 79.8 pg/ml in the adenine vs. control group, *P* < 0.0345, Supplementary Fig. 2H) and MCP-1 (1801.0 ± 1091.0 vs. 642.5 ± 374.2 pg/ml in the adenine vs. control group, *P* < 0.033, Supplementary Fig. 2M), which were significantly increased following the change of diet at week 10. Although IL-33 returned to normal values at week 18 (Supplementary Fig. 3H), MCP-1 remained significantly elevated at week 18 (2332.0 ± 645.1 vs. 1071.0 ± 425.8 pg/ml in the adenine vs. control group, *P* < 0.003, Fig. 5D). Moreover, the level of indoxyl sulfate, a uremic toxin involved in the induction of inflammation, was elevated in the adenine group (0.9 ± 0.2 vs. 0.6 ± 0.2 vs. mg/ml in the adenine vs. control group, *P* < 0.042, Fig. 5E). Finally, we observed a marked increase in the level of CD45-expressing immune cells in the LV of rats from the adenine group (42.7 ± 17.9 vs. 10.4 ± 3.4 cells in the adenine vs. control group, *P* < 0.012, Fig. 5F, G). These results highlight the rise in the level of markers of inflammation in circulation as well as the kidneys and the hearts of rats from the adenine group.

### The cardiac transcriptome is altered in rats with adenine-induced CKD

**Fig. 6:**
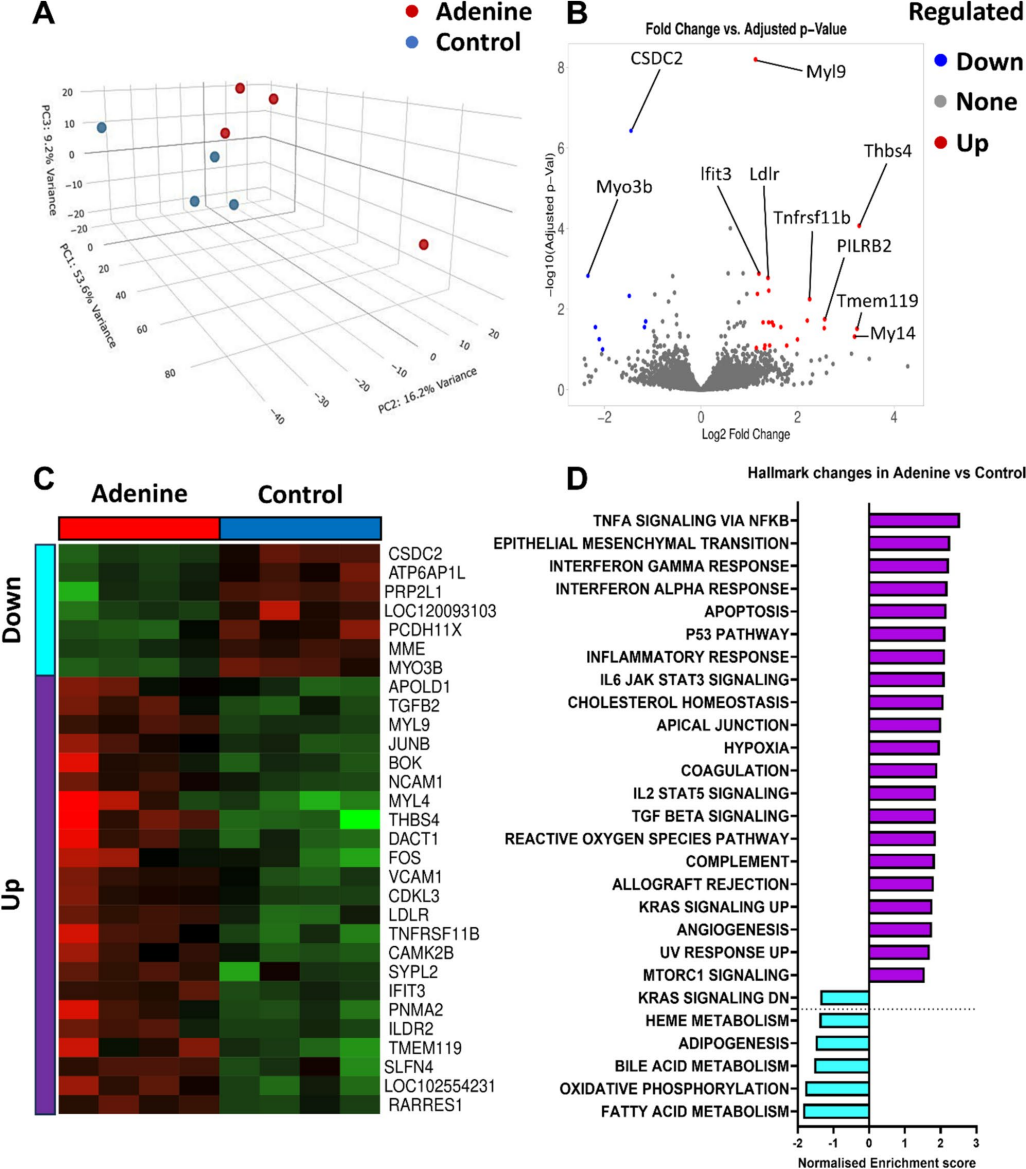
Cardiac transcription profile. Panel **A** shows the variability in gene expression among samples using principal components analysis. Differentially expressed genes are illustrated using a volcano plot in (**B**) and their expression profiles are illustrated using a heat map in panel (**C**). Alterations of Hallmark gene sets are shown in panel (**D**). (*n* = 4)

CKD has been shown to affect the transcriptomic profile of the heart [85]. To assess how CKD affect the cardiac transcriptomics in our model, we conducted RNA sequencing on LV samples obtained from rat hearts of the adenine group and control. Cardiac gene expression was significantly altered in the hearts of rats with CKD (Fig. 6A). Analysis of differentially expressed genes showed that 23 genes were significantly up-regulated and 7 genes were downregulated, respectively, in the hearts of rats from the adenine group in comparison to those from the control (Fig. 6B, C and Supplementary Table 2). Notable upregulated genes included transcription factors (*Fos*, *Junb* and *Tgfb2*) and cell adhesion proteins involved in inflammation (*Ncam1* and Vcam1), whereas genes involved in contraction (*Myo3b*) or cellular endocytosis (*Atp6ap1l*) were among genes that were significantly downregulated. Gene-set enrichment analysis using the “Hallmark” gene sets [48] showed an increase in the level of proinflammatory gene sets involved in the TNF-α pathway, apoptosis, reactive oxygen species, IL6 signaling and IL2 signaling (Fig. 6D and Supplementary Table 3). In comparison, gene sets relevant to cardiac metabolism such as fatty acid metabolism and bile acid metabolism were downregulated in the hearts of rats from the adenine group. In addition, gene sets involved in oxidative phosphorylation were downregulated (Fig. 6D), and this occurred in parallel to the reduction in citrate synthase (1.4 ± 0.1 vs. 1.7 ± 0.3 a.u. in the adenine vs. control group, *P* < 0.040, Supplementary Fig. 4A). Moreover, gene sets involved in the fibrosis-related epithelial mesenchymal transition (EMT) pathway were increased. Analysis at the level of individual genes also revealed an increase in the expression of *Acta1* (24,455 ± 18,856 vs. 10,819 ± 2038 read counts in the adenine vs. control group, *P* < 0.028, Supplementary Fig. 4C), *Tgfb1* (382.8 ± 29.3 vs. 302.3 ± 20.3 read counts in the adenine vs. control group, *P* < 0.0041, Supplementary Fig. 4G) and *Tgfb2* (1216.0 ± 468.3 vs. 459.0 ± 112.8 read counts in the adenine vs. control group, *P* < 0.02, Supplementary Fig. 4H) were significantly increased in hearts from the adenine group. No significant differences were observed in expression data for Vimentin, *Acta2*, *Col12a1*, *Col1a1*, or *Tgfb3* (Supplementary Fig. 4B, D, E, F, and I, respectively). Overall, these data indicate altered expression of genes participating in inflammation, fibrosis and metabolism in the LV tissue of rats from the adenine group.

### Adenine-induced CKD causes capillary rarefaction in the heart and increases the susceptibility of the heart to IR injury

**Fig. 7:**
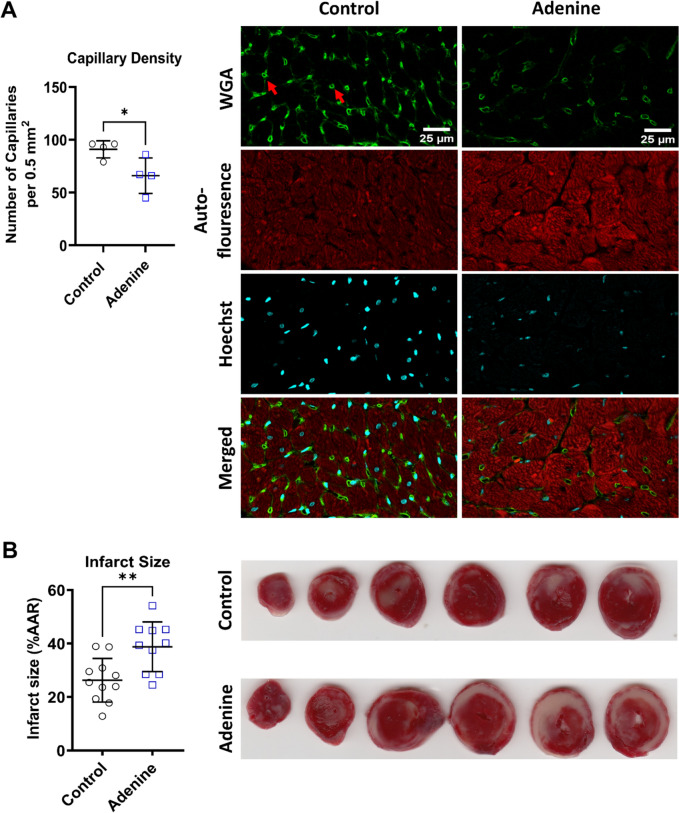
Changes in vascular density and response to ischemia–reperfusion injury. Panel **A** depicts the presence of rarefaction (capillaries are shown by red arrows) in the hearts of rats receiving the adenine diet (*n* = 4, *P < 0.05, Student's *t* test), whereas **B** shows the differences in cardiac infarct size (*n* = 11 for control and *n* = 10 for adenine, **P < 0.01, Student’s *t* test). (Data are expressed as mean ± SD)

CKD has been shown to be associated with microvascular rarefaction in patient hearts [77]. To investigate whether this occurs in our model, we assessed capillary density in rat hearts at the end of the study. There was a significant decrease in capillary density in the hearts of rats receiving dietary adenine (66.0 ± 16.7 vs. 91.0 ± 8.1 capillaries per 0.5 mm^2^ in the adenine vs. control group, *P* < 0.036, Fig. 7A). Analysis of the RNAseq data revealed that there was a significant reduction in angiopoietin 1 (*Angpt1)* (668.5 ± 224.3 vs. 1266.0 ± 267.2 read counts in the adenine vs. control group, *P* < 0.014, Supplementary Fig. 5A) but not angiopoietin 2 (*Angpt2)*. In addition, the ratio of *Angpt2* to *Angpt1* was significantly increased in the adenine group (0.4 ± 0.5 vs. 0.1 ± 0 in the adenine vs. control group, *P* < 0.028, Supplementary Fig. 5B and 5C). Further evaluation of ANGPT1 protein in the hearts by Western blot analysis showed no difference between the groups (Supplementary Fig. 5D). However, and intriguingly, ANGPT1 protein level was significantly reduced in the kidneys of adenine-fed rats (0.01 ± 0.00 vs. 0.04 ± 0.00 read counts in the adenine vs. control group, *P* < 0.0001, Supplementary Fig. 5E). CKD worsens the injury sustained from MI in human patients [20, 83]. In this context, the hearts from the adenine group had higher infarct size in comparison to the control group (38.8 ± 9.3% vs. 26.3 ± 8.2% in the adenine vs. control group, *P* < 0.003, Fig. 7B). Together, these findings suggest that adenine-induced CKD causes capillary rarefaction in the myocardium, which exacerbates the injury following IR.

## Discussion

In this study, we developed a modified adenine-induced model of CKD, in which rats were treated with a diet containing 0.3% adenine for 10 weeks before being returned to a normal diet for 8 weeks. In this model, we observed that the cardiac effects of CKD were maintained following the return to normal chow. The deterioration in renal function persisted even after the return to normal chow, indicating irreversible injury. Cardiac performance also declined in terms of ejection fraction and diastolic function during these 8 weeks. These changes occurred concurrently with systemic inflammation which may have contributed to the pathological kidney and cardiac function in rats from the adenine group. We also provided evidence for CKD-induced CVD (the so-called “cardio-renal” syndrome), with evidence of capillary rarefaction and increased sensitivity to IR injury in the hearts of rats in the adenine group.

The CKD model that was developed in this study has fewer limitations in terms of renal and cardiac pathologies in comparison to other models of CKD. For instance, unilateral ureteral obstruction (UUO) results in rapid kidney injury and a significant increase in LV mass as early as two weeks after the surgery. In addition, since UUO affects a single kidney, the compensatory function of the contralateral kidney may enhance or reduce the effects of UUO [44]. In comparison, 5/6 nephrectomy requires the removal of one kidney and a substantial portion of the remaining kidney. This ultimately causes a rapid hypertrophy of the remnant kidney due to a massive increase in pressure load and filtration [22, 44]. In addition, cardiovascular dysfunction as a result of 5/6 nephrectomy can be strain-specific and may require additional interventions for its induction [62, 85]. Our model on the other hand is based on a gradual progression of CKD that ultimately causes cardiovascular complications and fibrosis to closely mimic pathologies seen in human patients [12].

Our model shares some similarities with previous models of adenine-induced CKD, but also exhibits some key differences [15, 87]. This study was conducted on male rats, since the rate of cardiovascular-related mortality rate has been shown to be higher in men than women [57, 60, 72]. The observed physical characteristics of rats in our model are consistent with a previous report in which the rats’ body weight and food intake were reduced after 8 weeks of adenine administration [87]. Furthermore, a previous investigation by Diwan et al. reported an increase in BP of adenine-fed rats, whereas we did not observe any consistent changes in SBP or DBP in our experiments [15]. This may be due to the limitation of the tail-cuff techniques which underestimates BP, and may produce different readings in comparison to telemetry or invasive BP measurements [84]. Diminished kidney filtration capacity can contribute to hyperglycemia in patients with CKD [28]. In comparison to humans, a study in rats receiving a 0.25% adenine diet for 5 weeks found that rats had a higher fasting glucose [39]. However, we did not observe any difference in terms of blood glucose between groups, which may be due to the use of random blood sampling rather than the measurement of fasting blood glucose in our study.

In order to better understand the mechanism of CKD and identify potential drug targets, it is necessary to develop a model that closely mimics the pathologies seen in human patients. In this study, we chose the adenine model and aimed to enhance this model by allowing CKD and cardiac dysfunction to occur in the absence of adenine. In our model, the urinary albumin-to-creatinine ratio was elevated 8 weeks after returning to normal chow following the adenine diet. Interestingly, although the creatinine level was reduced from the pre-wash-out period, it remained significantly elevated at the end of the experiment thereby suggesting the persistence of CKD following the 8-week wash-out period. The slight recovery in plasma creatinine following the washout period indicates that adenine-induced kidney damage is partially reduced following withdrawal. This suggests that continued administration of the nephrotoxic adenine compound results in a model more reflective of "acute-on-chronic" kidney injury (ACKD), as opposed to a model of CKD [27]. In comparison, Shuvy et al. found that serum creatinine had returned almost to baseline in rats receiving a 0.75% adenine diet for 7 weeks followed by 12 weeks of recovery from the adenine diet [74]. However, they used Sprague Dawley rats which are known to develop age-related nephropathy thereby reducing their suitability for the study of adenine-induced CKD [24]. In addition, the use of 0.75% dietary adenine has been shown to cause substantial weight loss and rapid induction of CKD [90], which makes the rate of progression of the model less comparable to human CKD. The extreme weight loss can also necessitate premature termination of the experiment for ethical reasons.

The persistence of the CKD phenotype following the return to normal chow after the adenine diet has also been studied in a mouse model of CKD [41]. Mice that received 2 or 4 weeks of a 0.2% adenine diet developed severe renal failure accompanied by elevated urea and reduced creatinine clearance, which partially resolved 8 weeks after change of diet [41]. In addition, crystal depositions in the kidneys of these mice were significantly reduced and became only localized to the outer medulla region [41]. However, we observed crystal deposition in both the cortex and medulla of the kidneys of the rats from the adenine group after returning to normal chow for 8 weeks.

We also observed signs of aortic calcification in rats in the adenine group. This has been observed previously in Sprague Dawley rats, but resolved following the return from adenine diet to normal chow [74, 87]. In contrast, we noted that the aortic calcification persisted following the recovery from the adenine diet.

The above differences indicate that the cardiac and renal pathologies in adenine-induced CKD vary among strains and species and, importantly, appear to depend on the dose and duration of adenine administration [41, 74]. Considering the aforementioned differences, our model allows the study of progressive CKD since cardiac and specifically renal pathologies persist even after the change of diet.

In patients, CKD is associated with heart failure with preserved or reduced ejection fraction [51] which manifests as diastolic dysfunction [68, 73]. Consistent with this, we detected a significant reduction in the E/A ratio in our model, indirectly indicating the development of diastolic dysfunction. Interestingly, Kashioulis et al. saw no difference in the E/A ratio in rats fed with 0.5% adenine for 9 weeks. However, the authors reported a significant diastolic dysfunction as evidenced by the reduction of the motion of the mitral valve annulus measured by the E/e’ ratio, a parameter that we did not assess in our study [40]. It is also noteworthy to mention that since the E/A ratio declined significantly, only once rats were returned to normal chow, we believe that our model with a washout period is preferable for investigating the cardiac injury that occurs from CKD as opposed to ACKD [27]. We also observed a significant reduction in ejection fraction and elevation of circulating NT-proBNP, which can be a sign of mechanical stress in the heart [26]. Therefore, in comparison to Kashioulis et al., our data indicate the presence of early signs of heart failure and the progression of LV dysfunction over time [40]. Based on its utility, our model may be further developed to study different stages of heart failure in the setting of CKD.

Inflammation is one of the hallmarks of CKD [76] and anti-inflammatory therapies are now being tested to target CVD-associated CKD [65, 66]. However, the exact contribution that different inflammatory pathways make to the pathogenesis of CKD-associated CVD is not fully understood. To start to address this, we assessed the tissue and plasma levels of markers of inflammation that contribute to both cardiac and renal dysfunction. We demonstrated an increase in the level of indoxyl sulfate in the plasma of rats with CKD. Indoxyl sulfate is a gut-derived uremic toxin that is associated with the development of CVD in CKD patients [21]. It can also increase the level of several inflammatory markers such as NF-κB, MCP-1, IL6, and NLRP3 [88]. However, we did not assess the exact cause of the indoxyl sulfate increase or its contribution to the inflammation and cardiorenal outcomes. In line with previous reports, we observed a significant increase in NLRP3 and GSDMD proteins in the kidneys of rats receiving adenine. This may indicate the potential involvement of pyroptosis in the development of CKD [14, 53, 75]. The cell types contributing to the elevation of these proteins were not determined but are likely to be, at least partially, due to infiltrating inflammatory cells, which express high levels of these proteins [50]. However, we did not detect any changes in the level of circulating IL-1β, which has been shown to contribute to CVD-associated CKD in human patients [66]. It is possible that this would become more evident following the longer development of CKD.

KIM-1 is an important biomarker of CKD and is elevated in human patients with CKD [46]. KIM-1 overexpression has been shown to induce cardiac hypertrophy in mice and directly affects the level of MCP-1 [33]. In contrast to the mouse model of adenine-induced CKD, where circulating KIM-1 is reduced following the recovery from the adenine diet, we observed an elevated level of KIM-1 in our model thereby suggesting the presence of underlying kidney inflammation [41]. In line with the preceding analysis, we also detected elevated plasma MCP-1. MCP-1 has been shown to worsen renal and cardiac function in human and animal models of CKD [17, 69]. The levels of MCP-1 remained elevated throughout the 8 weeks of recovery from the adenine diet which emphasizes its importance in the induction of both cardiac and renal dysfunction during CKD. Similarly, we detected an elevation in the level of IL-33 in the adenine group following the change of diet. However unlike MCP-1, IL-33 levels were normalized toward the end of the study. IL33 is an inflammatory cytokine and its levels are inversely associated with decreased estimated glomerular filtration rate and positively correlate with adverse cardiovascular events in CKD patients [25]. Considering that IL-33 can cause cardiomyopathy following acute kidney injury [19], it is possible that it was causally involved in the induction of adverse cardiac function during the initiation phase of CKD in our model. Moreover, we observed an increase in the plasma level of MPO protein. It is likely that the plasma level of MPO contributed to the induction of cardiovascular dysfunction in our model since its elevation is known to be associated with vascular dysfunction in CKD [91]. In addition, elevated MPO is associated with increased incidence of heart failure and cardiovascular composite outcomes including MI in patients with CKD [35].

We extended our analysis on inflammation by assessing the transcriptomic profile of the hearts from different groups. Our evaluation showed the upregulation of numerous Hallmark gene sets involved in inflammation including TNFα signaling and IL-6, both of which are known to participate in the development of CKD-associated CVD in patients [36, 55]. Taken together, these findings demonstrate the relevance of our model to human CKD and prove its utility for studying the contribution of inflammation to the pathological kidney-heart crosstalk in the setting of CKD. Nevertheless, the mechanisms that lead to inflammatory cytokine production and transcriptomic profile in our model have not yet been established.

The patients with uremic CKD often present with myocardial fibrosis and cardiac hypertrophy [34, 56]. Although we did not detect maladaptive ventricular hypertrophy, our histological examination confirmed evidence for early signs of fibrosis in the adenine group. Our Hallmark gene set analysis confirmed an increase in gene sets involved in EMT. EMT contributes to fibrosis as it entails the transformation of epithelial cells into mobile mesenchymal cells and the subsequent generation of fibroblasts [82]. We also observed an increase in the expression level of mRNA for Tgf-β isoforms, which can facilitate the induction of EMT [82, 86]. However, the relevance of EMT to the induction of fibrosis and ventricular dysfunction in the setting of adenine-induced CKD-associated CVD remains to be investigated.

CKD both increases the risk of adverse cardiovascular events such as MI [10, 18] and worsens the outcome [20, 83]. It is therefore notable that in our experimental model, the hearts of adenine-fed rats were significantly more susceptible to IR injury. This suggests that the model can be used to investigate the mechanism by which CKD increases the susceptibility of the heart to IR injury and to identify potential targets to protect these hearts.

It is possible that the observed increase in myocardial infarct size following IR in the CKD rats is a consequence of altered mitochondrial energetics or changes in vascular density. In terms of mitochondrial function, we saw a decrease in mRNA pathways responsible for mitochondrial oxidative phosphorylation and content at the protein level, which have been shown to be pivotal for the development of uremic cardiomyopathy [79]. However, additional analysis is necessary to delineate the exact changes to mitochondrial function, for example in terms of ROS production [30], in the heart of rats with CKD. Furthermore, capillary rarefaction in the heart is common in patients with CKD [77]. Capillary rarefaction has also been reported in the heart of rats with adenine-induced CKD [1] and may stem from sympathetic nerve hyperactivity [2, 31]. Here, we found that capillary density is significantly reduced in the hearts of rats from the adenine group. Since capillary density correlates with infarct size [61], this suggests one clear mechanism by which CKD, via capillary rarefaction, may potentially increase the susceptibility of the heart to IR injury. Angiopoietin 1 (ANGPT1), is essential for the maintenance of vessel stability [54]. The ratio of circulating ANGPT2 to ANGPT1, has been shown to have prognostic value for the detection of kidney disease and heart failure [47, 54]. Although we did not observe a significant reduction of ANGPT1 protein in the heart, Angpt1 mRNA and protein levels were significantly downregulated in the heart and kidney of the adenine group, respectively. In addition, the mRNA ratio of Angpt2 to Angpt1 ratio was increased in the adenine group. Therefore, we speculate these changes affect the circulating level of Angpt1 and contribute to capillary rarefaction and adverse cardiac remodeling. However, this will need to be addressed in future studies. It is also important to note that the changes in the mRNA and protein expression were assessed in the entire heart and hence may not necessarily reflect changes at the level of individual cellular components [52]. Although we observed some changes indicating potential mechanism, a limitation is that we did not conduct any intervention studies, so at this point the study remains primarily descriptive. In addition to capillary rarefaction, a consequence of CKD in patients can be the “no-reflow” phenomenon following IR injury, which prevents complete reperfusion of the ischemic area [29, 43]. The occurrence of the no-reflow phenomenon is a strong predictor of death in CKD patients treated with primary percutaneous intervention [70]. Moreover, CKD can cause vascular dysfunction by decreasing nitric oxide bioavailability [58, 80, 81]. In our study, we observed significant capillary rarefaction in the heart, however, we did not assess vascular function and the no-reflow phenomenon. Further work will be required to assess their mechanistic role in the development of IR injury in our model.

In conclusion, we have shown that the pathological cardiac and renal effects of CKD persist following the change of the adenine diet in rats. This model is preferable to previously described adenine models since it allows the investigation of the chronic stage of kidney disease as well as its cardiovascular complications in the absence of ongoing direct injury caused by adenine. As such, it may be useful for future mechanistic studies of CKD-associated CVD.

## Supplementary Information

Below is the link to the electronic supplementary material.

Supplementary file1 (PDF 730 KB)

## Data Availability

The data is available upon reasonable request.
