# Supplementary material for: Investigating the cause of cardiovascular dysfunction in chronic kidney disease: capillary rarefaction and inflammation may contribute to detrimental cardiovascular outcomes
Source: Basic Res Cardiol. 2024 Oct 30;119(6):937–55. doi: 10.1007/s00395-024-01086-6 (PMC11628583; doi:10.1007/s00395-024-01086-6)
Supplement: Supplementary file 1 — Supplementary file1 (PDF 730 KB) [file 395_2024_1086_MOESM1_ESM.pdf]

Investigating the cause of cardiovascular dysfunction in chronic kidney disease: capillary rarefaction and inflammation may contribute to detrimental cardiovascular outcomes.

Basic Research in Cardiology

Siavash Beikoghli Kalkhoran[1], Maryna Basalay[1], Zhenhe He[1], Pelin Golforoush[1], Tayeba Roper[2], Ben Caplin[2], Alan D. Salama[2], Sean M Davidson[1], Derek M Yellon[1].

[1] The Hatter Cardiovascular Institute, University College London, 67 Chenies Mews, London WC1E 6HX, United Kingdom.

[2] University College London, Centre for Kidney and Bladder Health, Royal Free Hospital, London, England.

Corresponding author.

Professor Derek Yellon
Professor of Molecular & Cellular Cardiology
Director of the Hatter Cardiovascular Institute
University College London & UCL Hospital
67 Chenies Mews
London WC1E 6HX

Tel: +44 203 447 9591
PA: g.jarvis@ucl.ac.uk

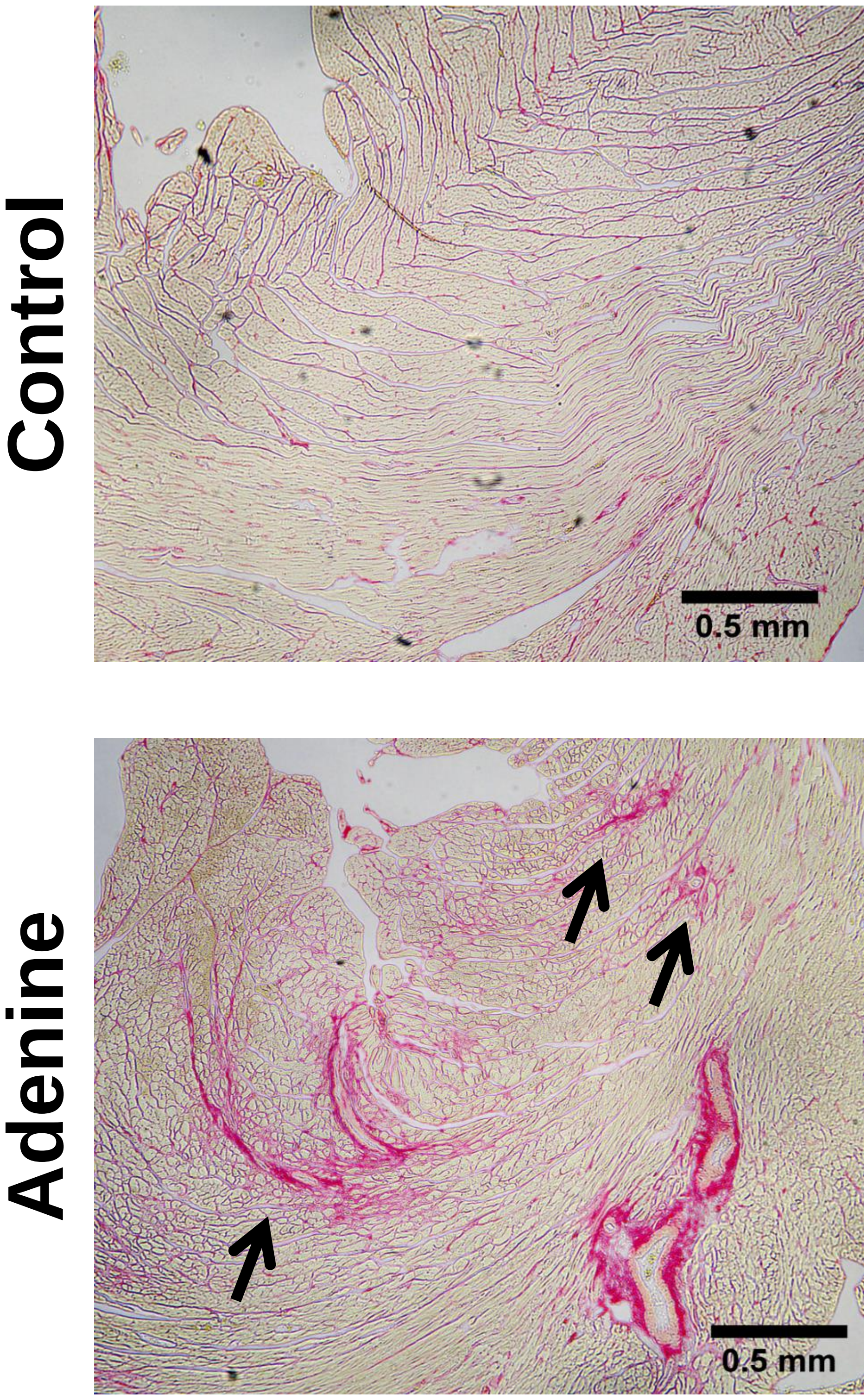

Supplementary Fig. 1. Picrosirius red staining of the LV. Areas of fibrosis can be seen in dark red staining indicated by black arrows.

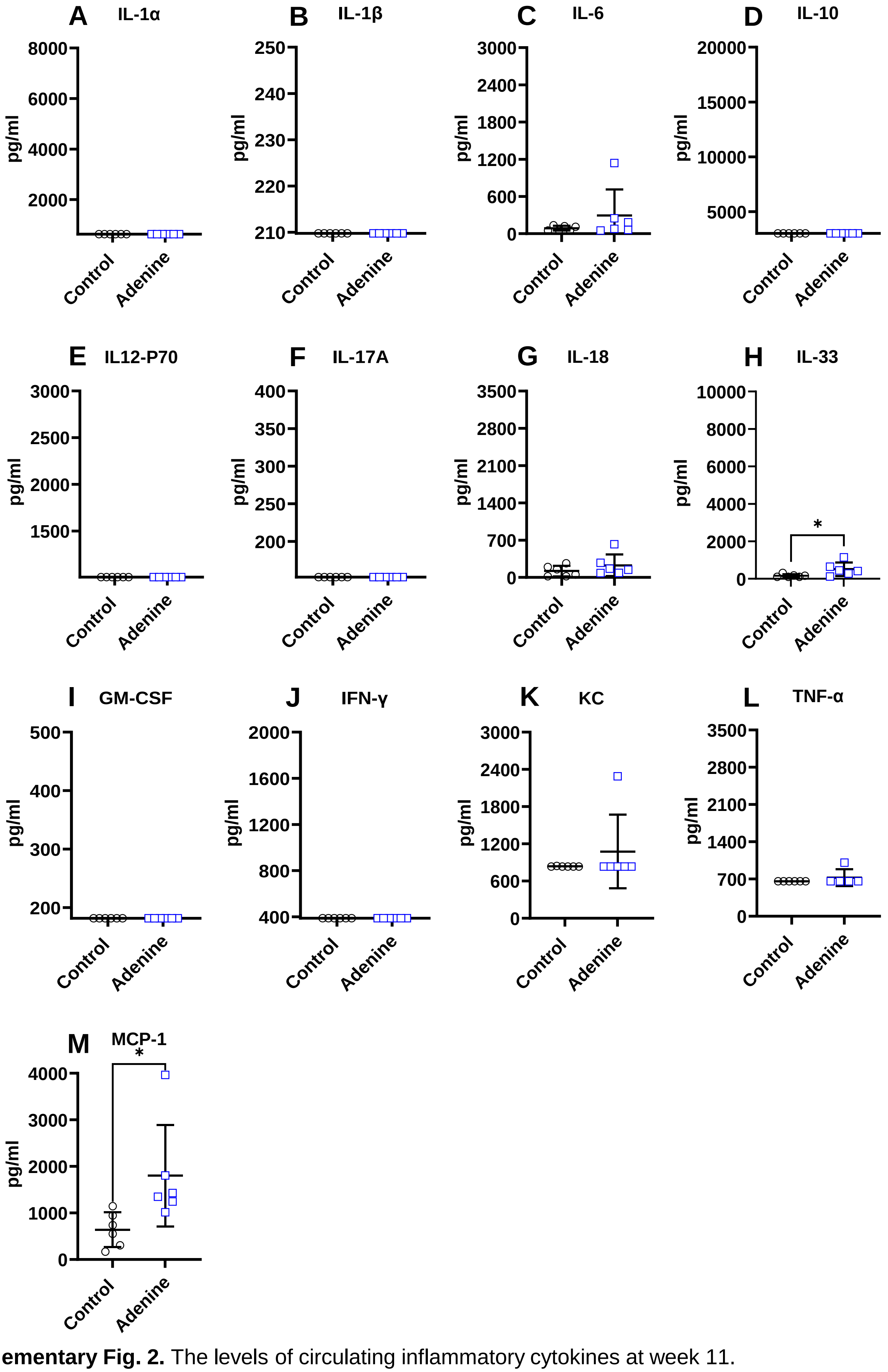

Supplementary Fig. 2. The levels of circulating inflammatory cytokines at week 11.
There data indicates the changes in protein levels of different inflammatory markers in the plasma of control and adenine-fed rats. All analysis was conducted using Student's t-test. (Data is expressed as mean ± SD).

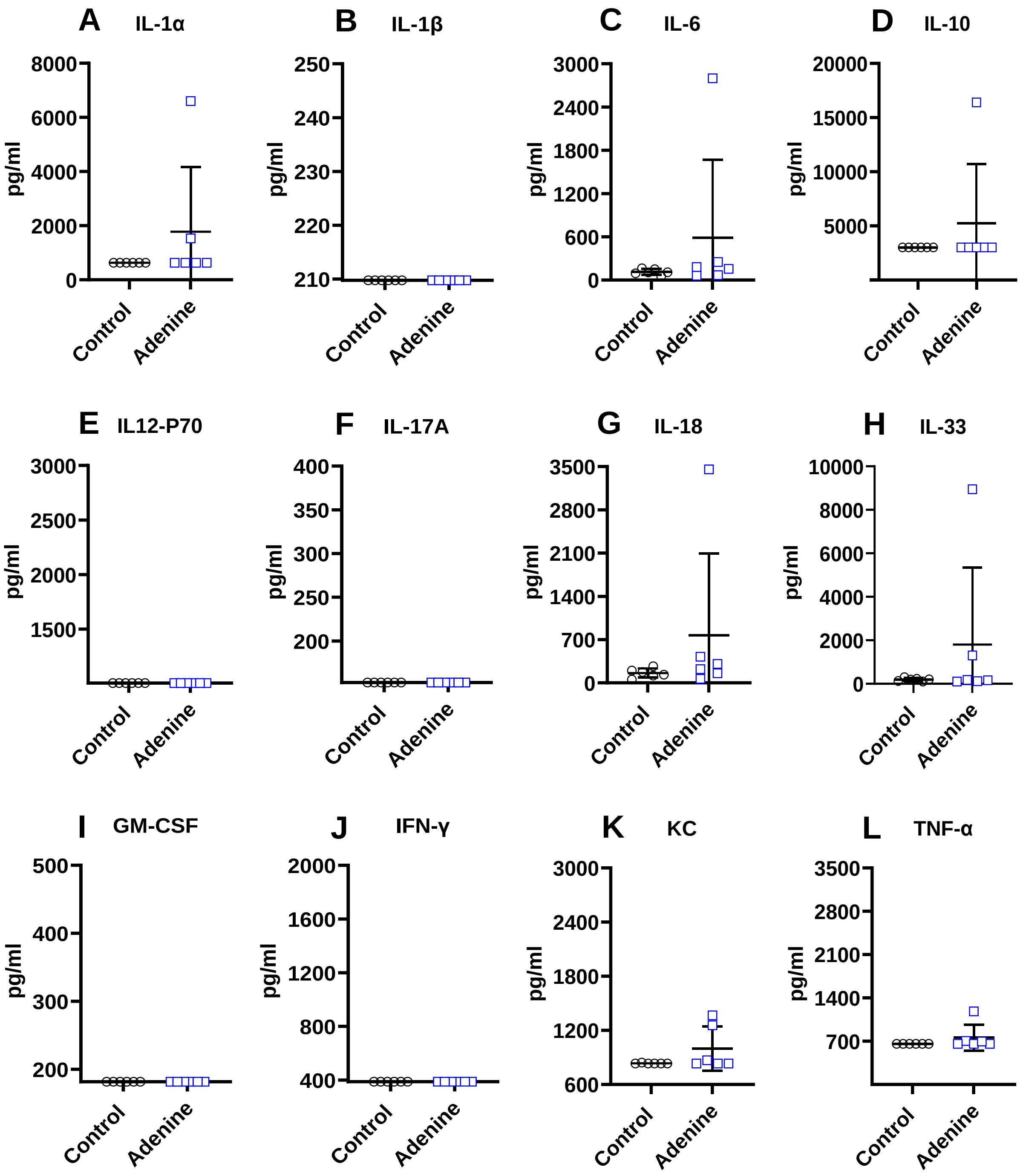

Supplementary Fig. 3. The levels of circulating inflammatory cytokines at week 18.
There data indicates the changes in protein levels of different inflammatory markers in the plasma of control and adenine-fed rats. All analysis was conducted using Student's t-test**.** (Data is expressed as mean ± SD).

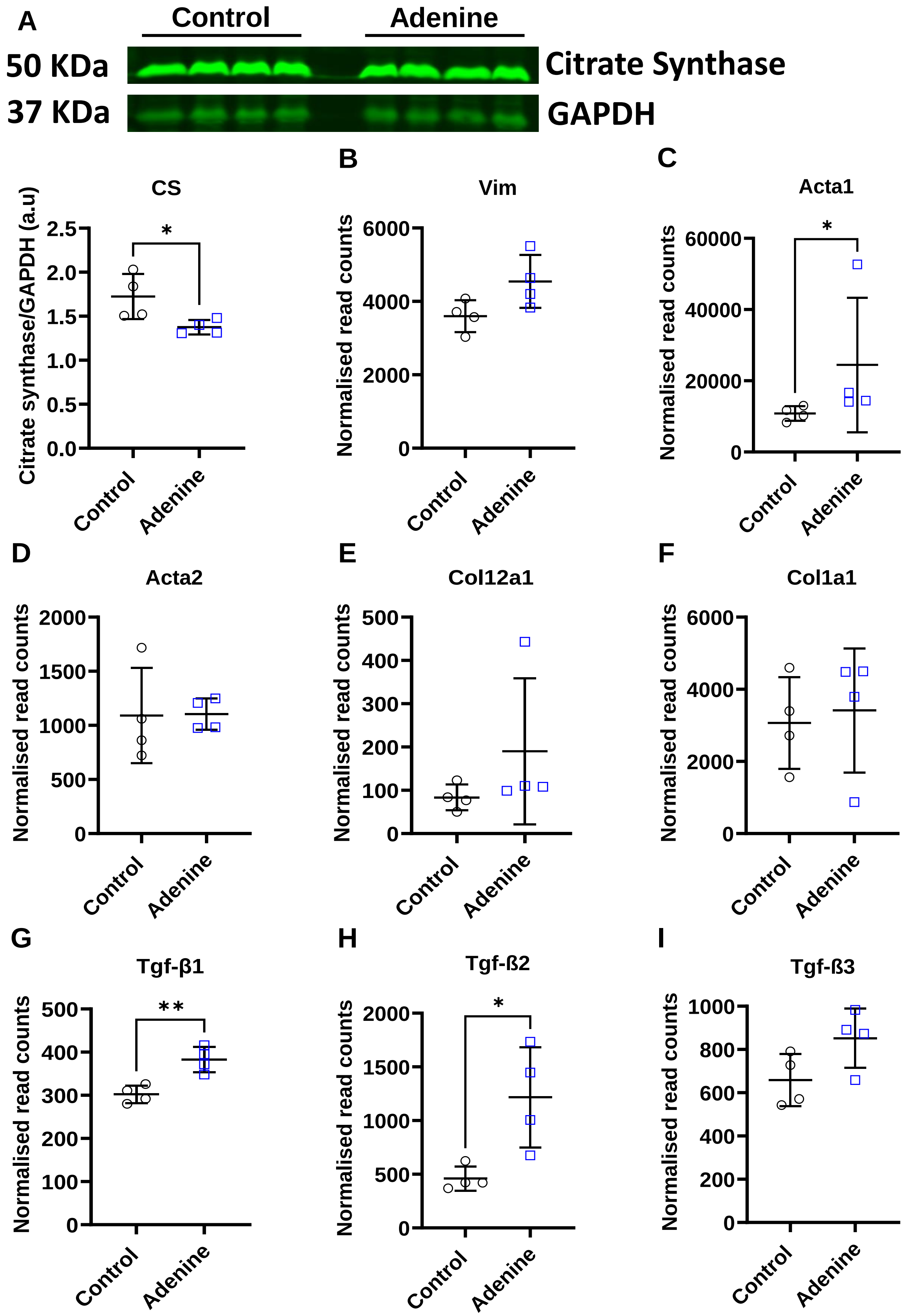

Supplementary Fig 4. Alteration of a mitochondrial protein and fibrosis-related genes in the adenine group.

(A) shows the protein level of citrate synthase in the heart of control versus adenine –fed rats. Normalised read counts of the mRNA expression from different genes involved in fibrosis are shown in **(B-I)**. Acta1 levels were analysed using Mann-Whitney U-test. All other analysis was conducted using Student's t-test. (Data is expressed as mean ± SD).

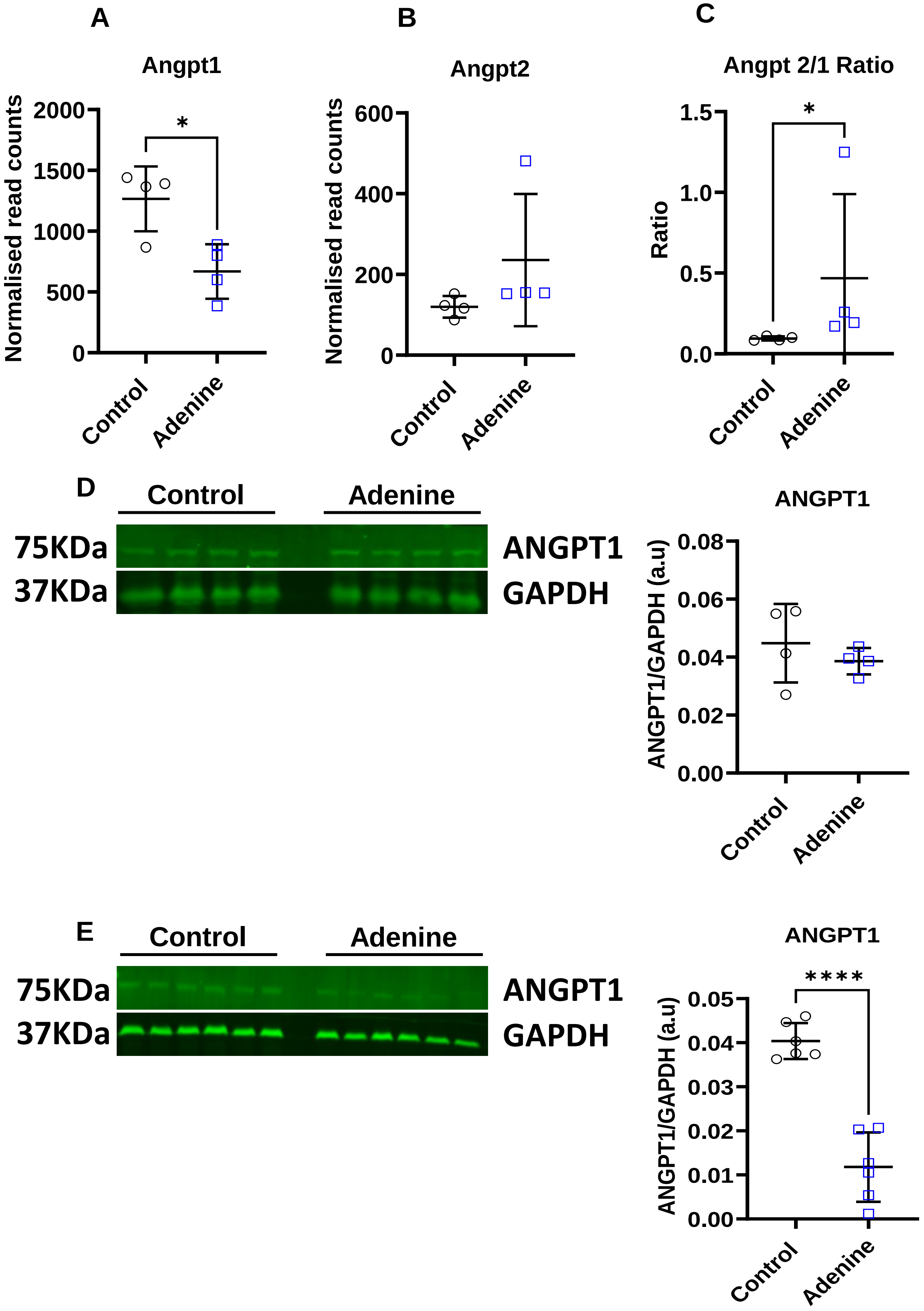

Supplementary Fig 5. Alteration of angiopoietin protein and genes in the heart and kidneys of rats with CKD.
(A) and **(B)** show the normalised read counts of mRNA level of Angpt1, Angpt2 and Angpt2/Angpt1 ratio in the heart of control versus adenine–fed rats, respectively. Protein levels of ANGPT1 in the heart and kidney from control and adenine group are shown in **(D)** and **(E)**, respectively. All analysis were conducted using Student's t-test. (Data is expressed as mean ± SD).

Supplementary Table 1. Outputs of two-way ANOVA analysis.

Figure number	Source of Variation	% of total variation	P value
Fig. 2A	Interaction	19.37	<0.0001
	Weeks	19.99	<0.0001
	Treatments	30.50	<0.0001
Fig. 2B	Interaction	6.99	<0.0001
	Weeks	52.72	<0.0001
	Treatments	28.00	<0.0001
Fig. 2C	Interaction	3.54	<0.0001
	Days	4.82	<0.0001
	Treatments	86.19	<0.0001
Fig. 2D	Interaction	3.57	0.1644
	Weeks	49.86	<0.0001
	Treatments	0.45	0.3161
Fig. 2E	Interaction	4.14	0.2599
	Weeks	17.02	0.0002
	Treatments	11.17	<0.0001
Fig. 2F	Interaction	3.22	0.1665
	Weeks	50.93	<0.0001
	Treatments	2.43	0.0155
Fig. 2G	Interaction	4.84	0.2468
	Weeks	16.47	0.0007
	Treatments	1.62	0.1347
Fig. 4A	Interaction	11.14	0.0058
	Weeks	19.87	<0.0001
	Treatments	0.00	0.9874
Fig. 4B	Interaction	2.35	0.5802
	Weeks	30.90	<0.0001
	Treatments	0.07	0.7391
Fig. 4C	Interaction	9.89	0.0056
	Weeks	13.90	0.0004
	Treatments	15.30	<0.0001
Fig. 4D	Interaction	1.06	0.8760
	Weeks	34.68	<0.0001
	Treatments	0.22	0.5437
Fig. 4E	Interaction	7.26	0.0589
	Weeks	21.64	<0.0001
	Treatments	0.00	0.9519

Supplementary Table 2. Statistical parameters of differentially expressed genes.

Gene symbol and Loci	log2 Fold Change	Adjusted P value
**Thbs4**	3.28	9.58E-05
**Tmem119**	3.22	3.02E-02
**Myl4**	3.19	4.85E-02
**LOC102554231**	2.55	2.14E-02
**Pnma2**	2.55	3.02E-02
**Tnfrsf11b**	2.26	5.73E-03
**Dact1**	2.20	1.89E-02
**Fos**	2.00	5.61E-02
**Bok**	1.77	8.01E-02
**Camk2b**	1.65	2.78E-02
**Ildr2**	1.50	2.52E-02
**Rarres1**	1.47	2.14E-02
**Slfn4**	1.42	7.98E-02
**Tgfb2**	1.40	3.45E-03
**Junb**	1.40	2.14E-02
**Ldlr**	1.40	1.79E-03
**Apold1**	1.32	7.91E-02
**Sypl2**	1.32	9.37E-02
**Vcam1**	1.29	2.14E-02
**Ifit3**	1.20	1.31E-03
**Ncam1**	1.17	4.27E-03
**Cdkl3**	1.15	9.26E-02
**Myl9**	1.13	6.64E-09
**Myo3b**	-2.32	1.31E-03
**LOC120093103**	-2.19	2.78E-02
**PCDH11X**	-2.11	5.56E-02
**PRP2L1**	-1.49	4.70E-03
**Csdc2**	-1.43	3.69E-07
**Atp6ap1l**	-1.17	2.78E-02
**Mme**	-1.15	2.06E-02

Supplementary Table 3. Statistical parameters of Hallmark pathways analysis.

Pathway	Adjusted P Value	Normalised Enrichment score	Size of gene set
**TNFA SIGNALING VIA NFKB**	2.29E-18	2.540235	187
**EPITHELIAL MESENCHYMAL TRANSITION**	3.61E-12	2.268322	182
**INTERFERON GAMMA RESPONSE**	2.46E-11	2.234137	187
**INTERFERON ALPHA RESPONSE**	1.39E-07	2.194609	95
**APOPTOSIS**	1.05E-09	2.164746	156
**P53 PATHWAY**	1.06E-09	2.135754	189
**INFLAMMATORY RESPONSE**	1.25E-09	2.12858	189
**IL6 JAK STAT3 SIGNALING**	1.98E-06	2.114865	82
**CHOLESTEROL HOMEOSTASIS**	1.58E-05	2.085405	71
**APICAL JUNCTION**	5.91E-08	2.015113	190
**HYPOXIA**	1.55E-07	1.978497	186
**COAGULATION**	1.58E-05	1.91081	113
**IL2 STAT5 SIGNALING**	4.87E-06	1.869762	190
**TGF BETA SIGNALING**	0.000979	1.865311	51
**REACTIVE OXYGEN SPECIES PATHWAY**	0.002003	1.865106	48
**COMPLEMENT**	2.27E-05	1.840493	174
**ALLOGRAFT REJECTION**	8.78E-06	1.813164	184
**KRAS SIGNALING UP**	2.25E-05	1.775194	186
**ANGIOGENESIS**	0.005872	1.760716	31
**UV RESPONSE UP**	0.000272	1.703807	154
**MTORC1 SIGNALING**	0.003725	1.55341	195
**KRAS SIGNALING DN**	0.030263	-1.36589	153
**HEME METABOLISM**	0.018298	-1.39028	186
**ADIPOGENESIS**	0.003725	-1.49645	194
**BILE ACID METABOLISM**	0.00981	-1.53655	107
**OXIDATIVE PHOSPHORYLATION**	1.58E-05	-1.78335	197
**FATTY ACID METABOLISM**	2.39E-05	-1.84328	150
